# Characterisation of non-degraded oligosaccharides in enzymatically hydrolysed and fermented, dilute ammonia-pretreated corn stover for ethanol production

**DOI:** 10.1186/s13068-017-0803-3

**Published:** 2017-05-02

**Authors:** M. C. Jonathan, J. DeMartini, S. Van Stigt Thans, R. Hommes, M. A. Kabel

**Affiliations:** 1 Laboratory of Food Chemistry, Wageningen University & Research, Bornse Weilanden 9, 6708 WG Wageningen, The Netherlands; 2 DuPont Industrial Biosciences (Genencor Division), 925 Page Mill Road, Palo Alto, CA 94304 USA; 3 DuPont Industrial Biosciences (Genencor International B.V.), Archimedesweg 30, 2333 CN Leiden, The Netherlands

**Keywords:** Corn stover, Glucuronamide, Hexenuronic acid, Hexenuronamide, Aldouronic acid, Xyloglucan, Xylan

## Abstract

**Background:**

Corn stover is lignocellulosic biomass that has potential to be used as raw material for bioethanol production. In the current research, dilute ammonia pretreatment was used to improve the accessibility of corn stover carbohydrates to subsequently added hydrolytic enzymes. Some carbohydrates, however, were still present after enzymatic hydrolysis and fermentation. Hence, this research was aimed to characterise the recalcitrant carbohydrates, especially the oligosaccharides that remained after hydrolysis and fermentation of dilute ammonia-pretreated corn stover (DACS).

**Results:**

About 35% (w/w) of DACS carbohydrates remained after enzymatic hydrolysis and fermentation of the released monosaccharides. One-third of these recalcitrant carbohydrates were water soluble and composed of diverse oligosaccharides. By using UHPLC-MS^*n*^, more than 50 oligosaccharides were detected. Glucurono-xylooligosaccharides (UAXOS) with a degree of polymerisation (DP) less than 5 were the most abundant oligosaccharides. The (4-*O*-methyl) glucuronosyl substituent was mostly attached onto the terminal xylosyl residue. It was shown that the glucuronosyl substituent in some UAXOS was modified into a hexenuronosyl, a glucuronamide or a hexenuronamide residue due to the dilute ammonia pretreatment. Another group of abundant oligosaccharides comprised various xyloglucan oligosaccharides (XGOS), with a DP 5 annotated as XXG as the most pronounced. In addition, disaccharides annotated as xylosyl-glucose with different β linkages as well as larger carbohydrates were present in the fermentation slurry.

**Conclusions:**

Around one-third of the 35% (w/w) recalcitrant DACS carbohydrates remained as water-soluble saccharides. In this study, more than 50 recalcitrant oligosaccharides were detected, which mostly composed of xylosyl and/or glucosyl residues. The most pronounced oligosaccharides were UAXOS and XGOS. Hence, α-glucuronidase and α-xylosidase were suggested to be added to the enzyme mixture to degrade these oligosaccharides further, and hence the fermentation yield is potentially increased.

**Electronic supplementary material:**

The online version of this article (doi:10.1186/s13068-017-0803-3) contains supplementary material, which is available to authorized users.

## Background

Corn stover is a lignocellulosic biomass that is abundantly available and used for bioethanol production. Based on dry weight, corn stover contains 30% (w/w) cellulose, 26% (w/w) hemicellulose, 4% (w/w) protein and 29% (w/w) lignin [1]. Like other lignocellulosic materials, pretreatment of corn stover is necessary to make the cellulose and hemicellulose accessible for the subsequently added hydrolytic enzymes [2–4]. The advantages and disadvantages of various pretreatment methods have been reviewed in detail [5]. In the current research, dilute ammonia pretreatment was used, which is known to release hemicelluloses from carbohydrate–lignin complex and the ammonia can be recycled [4].

Another important factor in biomass conversion is the composition of the enzyme cocktail used to hydrolyse the carbohydrate to monosaccharides. The choice of enzymes included in the cocktail depends on the structure of the carbohydrates present in the biomass. In corn stover, the major carbohydrates are cellulose and hemicellulose [1], with heteroxylan as the major hemicellulose [6]. The heteroxylan is composed of a xylan backbone substituted by arabinosyl residues, (4-*O*-methyl)-glucuronic acid residues and acetyl esters. The degree of substitution was reported to be on average 14 arabinosyl residues, 9 glucuronic acid residues and 39 acetyl esters for every 100 xylosyl residues [7]. In addition, the heteroxylan in corn stover contains ferulic and coumaric acid esters [7]. Hence, enzyme cocktails to hydrolyse corn stover carbohydrates should include endoglucanases, cellobiohydrolases and β-glucosidases to degrade the cellulose, as well as endoxylanase, β-xylosidase and various accessory enzymes to degrade the heteroxylan [8, 9]. To further optimise existing enzyme cocktails, information about the structures that remain after pretreatment, hydrolysis and fermentation is required. Such research has been conducted for dilute acid-pretreated corn fibre. It was found in the latter research that the recalcitrant oligosaccharides were heavily substituted xylooligosaccharides, many of which also contain acetyl and feruloyl esters [10, 11]. Corn stover, however, has a different composition from corn fibre [1]. Also, it has been shown that the type of pretreatment affects the type of remaining structures for corn stover degradation [12]. Alkaline pretreatments release the ester-linked substitutions such as acetyl and feruloyl esters [13]. Hence, the recalcitrant structures and, therefore, the accessory enzymes needed to degrade the recalcitrant structures from alkaline-pretreated corn stover were expected to be different from those for dilute acid-pretreated corn fibre. This research was aimed to characterise recalcitrant oligosaccharides after hydrolysis and fermentation of dilute ammonia-pretreated corn stover (DACS).

## Results

### Carbohydrate composition and identification of oligosaccharides in DACS

The carbohydrates in DACS were composed of mainly glucosyl and xylosyl residues (Table 1). Less than 15% (w/w) of the carbohydrates were soluble in water, but no oligosaccharides were detected in the water-soluble fraction (sDACS) when analysed by MALDI-TOF MS. After enzymatic hydrolysis and fermentation, about one-third of the carbohydrates present in DACS remained and the other two-thirds were converted to ethanol. In DACS after enzymatic hydrolysis and fermentation (F-DACS), more than 30% (w/w) of the carbohydrates were present in the water-soluble fraction (Fs-DACS). These soluble carbohydrates were enriched in xylosyl, arabinosyl and uronosyl residues (Table 1). This research was focused on the characterisation of these water-soluble recalcitrant carbohydrates.

**Table 1:** Constituent monosaccharide composition of dilute ammonia-pretreated corn stover before (DACS) and after enzymatic hydrolysis and fermentation (F-DACS)

Sample	Yield % (w/w)^a^	% mol							mol/mol	
		rha	ara	xyl	man	gal	glc	UA	ara/xyl	UA/xyl
DACS	100	0	6	37	0	2	51	3	0.17	0.09
iDACS^b^	83	0	4	34	0	1	59	2	0.11	0.07
sDACS^c^	13	1	19	51	1	7	11	10	0.36	0.19
F-DACS										
Fi-DACS^b^	21	0	3	30	1	1	61	3	0.11	0.11
Fs-DACS^c^	12	1	14	43	3	9	16	14	0.35	0.32

*rha* rhamnose, *ara* arabinose, *xyl* xylose, *man* mannose, *gal* galactose, *glc* glucose, *UA* uronic acids

^a^Yield was expressed based on the total carbohydrate present in DACS

^b^Water-insoluble fraction

^c^Water-soluble fraction

**Fig. 1:**
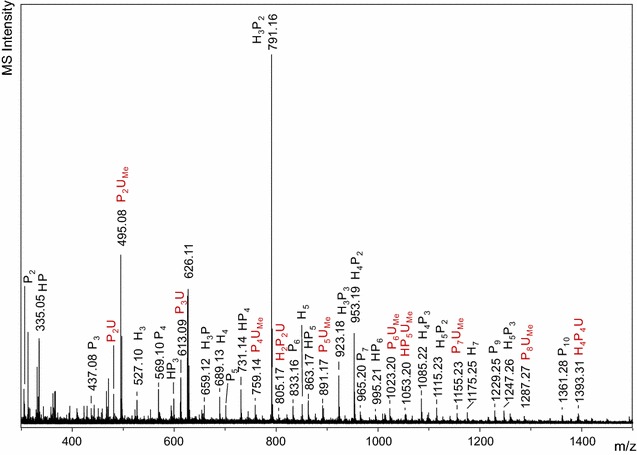
MALDI-TOF mass spectrum of the water-soluble fraction after enzymatic hydrolysis and fermentation of DACS (Fs-DACS). The ions were detected in positive mode as their sodium adducts (M + 23)^+^. *H* hexose, *P* pentose, *U* uronic acid, *U*_*me*_ 4-*O*-Me-uronic acid

The MALDI-TOF mass spectrum of Fs-DACS showed that the sample contained various oligosaccharides (Fig. 1), but no acetylated or hydroxycinnamic acid ester-containing oligosaccharides were detected. The oligosaccharide with the highest signal was annotated as a pentasaccharide (H_3_P_2_, *m*/*z* 791). Other oligosaccharides that were annotated as H_*x*_P_*y*_ and those indicative of degradation products of heteroxylan, such as HP_*n*_, P_*n*_UA, P_*n*_UA_me_ (*n* = 2–8), were also present.

### Separation of oligosaccharides by SPE C18 and isolation in 67% (v/v) ethanol

MALDI-TOF MS provided a fast method to obtain the oligosaccharide profile of Fs-DACS, but there was no distinction between isomers or between different hexoses or pentoses. To enable more detailed characterisation of the oligosaccharides, Fs-DACS was fractionated by means of C18 solid-phase extraction (SPE). The more polar oligosaccharides, including glucurono-xylooligosaccharides (UAXOS), were eluted with water (F0) and the less polar oligosaccharides, which were mainly annotated as H_*x*_P_*y*_ and P_*n*_ were eluted with 30% (v/v) methanol (F30) (Additional file 1: Figure S1A).

**Fig. 2:**
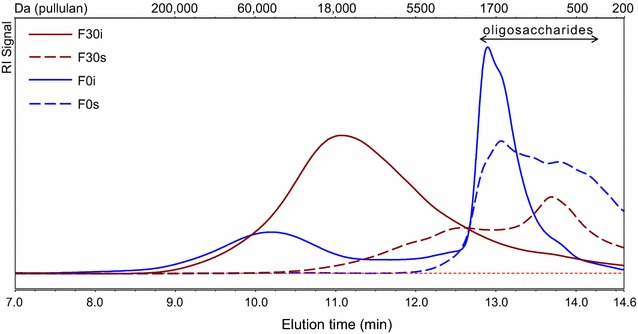
Molecular mass distribution of fractions obtained from F-DACS after C18 SPE and precipitation in 67% (v/v) ethanol, analysed using HPSEC-RI. F0s and F30s were 67% (v/v) ethanol-soluble fractions, F0i and F30i were 67% (v/v) ethanol-insoluble fractions

The oligosaccharides in F0 and F30 were isolated further by dissolution in 67% (v/v) ethanol, in which the large molecules precipitated. All the oligosaccharides in F0 and F30 were isolated in F0s and F30s, respectively, as shown by the molecular mass distribution (Fig. 2) and MALDI-TOF MS (Additional file 1: Figure S1A). The constituent monosaccharide composition of the 67% (v/v) ethanol-soluble (F0s and F30s) and ethanol-insoluble (F0i and F30i) fractions is presented in Table 2. In F0s, three quarters of the carbohydrates were composed of xylosyl (52%) and glucosyl (25%) residues. In F30s, the dominant constituents were xylosyl, glucosyl and arabinosyl residues.

**Table 2:** Constituent monosaccharide composition of Fs-DACS fractions after separation by C18 SPE and precipitation in 67% (v/v) ethanol

Fraction	% mol/mol									ara/xyl	glcA/xyl
	fuc	rha	ara	xyl	man	gal	glc	galA	glcA		
F0s	0	0	5	52	5	3	25	3	7	0.10	0.13
F0i	1	5	20	29	6	20	7	7	5	0.70	0.18
F30s	0	1	20	46	2	9	19	1	2	0.43	0.05
F30i	0	2	27	39	3	16	6	2	3	0.70	0.08

*rha* rhamnose, *ara* arabinose, *xyl* xylose, *man* mannose, *gal* galactose, *glc* glucose, *galA* galacturonic acid, *glcA* glucuronic acid, *F0* water-eluted fractions, *F30* 30% (v/v) methanol-eluted fractions, *s* 67% (v/v) ethanol-soluble fractions, *i* 67% (v/v) ethanol-insoluble fractions

F0i and F30i were rich in arabinosyl, xylosyl and galactosyl residues (Table 2). Using HPSEC (Fig. 2), it was shown that F0i and F30i contain large (>10 kDa) compounds. Analysis results on the glycosidic linkage composition of both F0i and F30i (Table 3) indicated the presence of highly substituted arabinoxylan, shown by the abundance of 3,4-linked and 2,3,4-linked xylosyl residues as well as terminal arabinosyl (t-ara) residues. Arabinan was present, indicated by the presence of arabinosyl residues with various glycosidic linkages. In addition, F0i may contain pectin, as shown by the presence of 2,4-linked rhamnosyl residue and the higher abundance of galacturonosyl residue compared with F30i (Table 3). F0i and F30i were not analysed further in this research.

**Table 3:** Glycosidic linkage composition of Fs-DACS fractions after separation by C18 SPE and isolation in 67% (v/v) ethanol

Linkage	mol%										
	F0s	F0i	F30s	F30i	SEC pools from F0s				SEC pools from F30s		
					A1–A2	A3–A7	A8–A13	A14–A15	B1–B6	B7–B14	B15–B17
2,4-rha	0	1	0	0	1	0	0	0	0	0	0
Total rha	0	1	0	0	1	0	0	0	0	0	0
t-ara	2	13	11	14	8	2	1	1	18	7	2
2-ara	1	6	4	8	1	0	2	0	6	4	1
3-ara	1	5	2	5	1	1	1	0	4	2	0
5-ara	0	7	3	4	6	0	0	1	5	1	0
2,5-ara	1	2	2	3	3	2	0	0	3	2	0
3,5-ara	0	1	0	0	0	0	0	0	0	0	0
2,3,5-ara	0	1	0	0	0	0	0	0	0	0	0
Total ara	4	34	23	33	19	6	4	3	36	16	4
t-xyl	24	10	21	11	6	10	19	46	12	19	33
2-xyl	10	6	6	5	4	19	7	8	5	8	4
3-xyl	1	1	1	1	0	0	2	2	1	2	2
4-xyl	21	7	16	11	13	43	21	4	14	18	10
2,3-xyl	0	1	1	1	1	1	0	0	2	1	1
3,4-xyl	1	11	6	16	3	3	0	0	11	5	0
2,3,4-xyl	0	7	3	8	1	1	0	0	6	2	0
Total xyl	58	41	55	53	29	77	50	59	50	54	50
t-man	3	1	0	0	1	2	4	3	0	0	0
4-man	1	0	0	0	0	0	1	1	0	0	0
Total man	4	1	0	0	1	2	5	4	0	0	0
t-gal	2	9	3	7	3	1	2	1	4	3	1
3-gal	0	2	0	1	0	0	0	0	1	1	0
4-gal	0	2	0	1	1	0	0	0	1	0	0
6-gal	0	1	0	1	1	0	0	0	0	0	0
Total gal	2	14	4	9	5	1	3	1	6	4	2
t-glc	9	2	3	1	5	3	9	12	1	6	2
2-glc	6	1	1	0	0	1	6	9	0	1	0
3-glc	1	0	0	0	0	0	1	1	0	0	0
4-glc	8	1	4	0	1	1	9	6	1	6	10
6-glc	6	2	5	1	34	7	12	5	4	8	20
3,6-glc	0	0	0	0	1	0	0	0	0	0	0
4,6-glc	0	0	3	0	0	0	1	0	0	3	11
Total glc	31	6	16	3	42	12	37	33	7	25	43
Total	99	96	99	98	97	99	99	99	98	99	100

Only neutral glycosyl residues were analysed. A glycosyl residue at the reducing end resulted in the same partially methylated alditol acetates as an internal glycosyl residue

*t* terminal, *rha* rhamnose, *ara* arabinose, *xyl* xylose, *man* mannose, *gal* galactose, *glc* glucose

### Structural elucidation of water-soluble, highly polar disaccharides and oligosaccharides in F0s

**Fig. 3:**
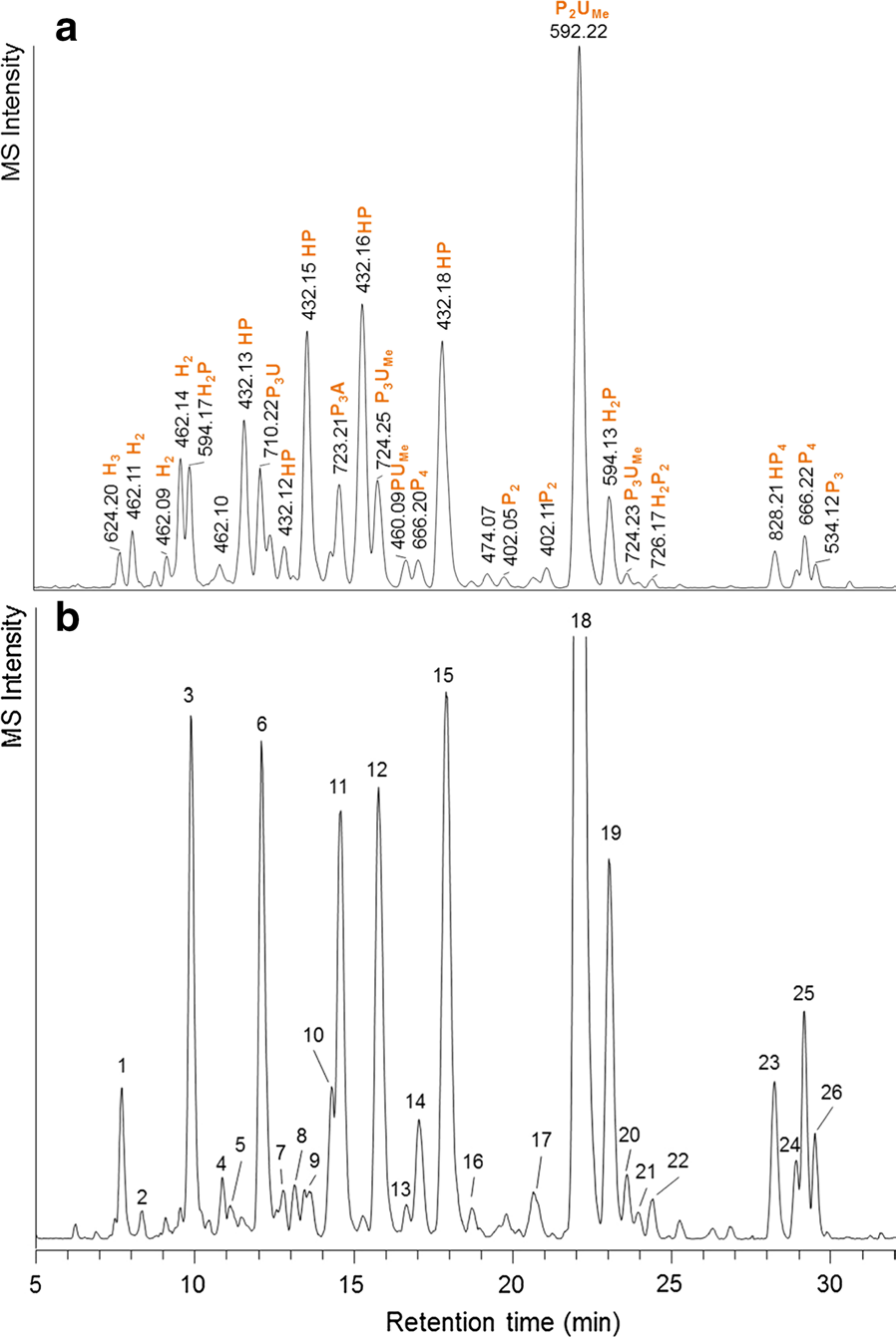
UHPLC-MS base-peak chromatogram of 2-AA-labelled F0s (*m*/*z* 400–2000) (**a**) and extracted ion chromatogram of oligosaccharides (*m*/*z* 501–2000) in 2-AA-labelled F0s (**b**). The peak numbers correspond to the data in Table 5

Structural elucidation of disaccharides and oligosaccharides in F0s was performed using UHPLC-MS^*n*^ after labelling the oligosaccharides using 2-aminobenzoic acid (2-AA) (Fig. 3a). The oligosaccharide (DP > 3) profile of F0s was obtained by extraction of peaks having *m*/*z* values between 501 and 2000 (Fig. 3b).

Figure 3a shows that some isomeric compounds were separated, so that the structures of each isomer could be investigated based on its MS^2^ fragmentation pattern. Nevertheless, the types of hexoses or pentoses were still unknown. Hence, F0s was fractionated further using size exclusion chromatography (SEC) and the pools obtained were analysed for their monosaccharide constituent (Table 4; Additional file 3: Table S1) and glycosidic linkage composition (Table 3; Additional file 4: Table S2). By combining the data obtained from the different analyses, the carbohydrates in F0s can be categorised as large molecules (SEC A1–A2), UAXOS (SEC A3–A7), neutral oligosaccharides (SEC A8–A13) and disaccharides (SEC A14–A15).

**Table 4:** Constituent monosaccharide composition of combined SEC pools obtained from F0s and F30s

Combined SEC pools^a^	% mol/mol								
	fuc	ara	rha	gal	glc	xyl	man	galA	glcA^b^
F0s									
A1–A2	0	12	4	10	30	29	3	7	5
A3–A7	0	4	0	2	12	64	4	3	10
A8–A13	0	5	0	4	32	45	5	2	8
A14–A15	0	5	0	2	32	54	4	1	1
F30s									
B1–B6	0	29	2	12	8	43	2	1	3
B7–B14	1	14	0	8	24	50	1	1	1
B15–B17	0	6	0	4	38	49	1	0	1

*fuc* fucose, *ara* arabinose, *rha* rhamnose, *gal* galactose, *Glc* glucose, *xyl* xylose, *man* mannose, *galA* galacturonic acid, *glcA* glucuronic acid

^a^SEC pools were combined based on their oligosaccharide profiles analysed by MALDI-TOF MS. The composition of combined pools were obtained from calculation based on the composition of individual pools

^b^Including 4-*O*-methyl-glucuronic acid

#### Large molecules (SEC A1–A2)

No oligosaccharides were detected by MALDI-TOF MS in SEC A1–A2. By HPSEC (Additional file 2: Figure S2), it was shown that these pools contained large molecules (1500–6000 Da, based on pullulan standards). The carbohydrates in these pools were rich in glucosyl and xylosyl residues (Table 4) and were enriched in arabinosyl, galactosyl and galacturonosyl residues compared with other pools from F0s. The relatively high amount (7% mol/mol) of galacturonosyl residue compared with the other SEC pools (Table 4) and the presence of 2,4-rha (Table 3) indicated the presence of pectin. Arabinan and arabinoxylan were present, shown by the abundance of 5-ara, t-ara and 4-xyl (Table 3). As much as 80% of the glucosyl residues were 1,6-linked.

#### Glucurono-xylooligosaccharides (UAXOS, SEC A3-A7)

Based on MALDI-TOF MS (not shown), pools A3–A7 were rich in pentosyl oligosaccharides containing an uronosyl residue. Indeed, the glycosidic linkage composition (Table 3) and the constituent monosaccharide composition (Table 4) of combined pools A3–A7 showed that 4-linked xylosyl residues and glucuronosyl residues were abundant. The most abundant oligosaccharide in F0s was P_2_U_me_ (*m*/*z* 592, peak 18, Fig. 3b), which was also identified by MALDI-TOF MS (*m*/*z* 495; Additional file 1: Figure S1B(i)). Based on the MS^2^ fragmentation analysis [14], the 4-*O*-Me-glucuronosyl residue was located at the terminal xylosyl residue and the compound could be annotated as U^4m2^X according to heteroxylan nomenclature [15]. Similarly, other UAXOS with a (4-*O*-Me)-glucuronosyl residue located at the terminal xylosyl residue (peaks 6, 12 and 15; Fig. 3b; Table 5) were present. The uronosyl substitution at *O*-2 of the terminal xylosyl residue was supported by the glycosidic linkage composition of combined pools A3–A7 (Table 3), which shows that about 25% of the xylosyl residues were 2-linked. Other isomers containing three pentosyl residues and an uronosyl substituent were present in small amounts (peaks 4, 8, 14, 16 and 20; Fig. 3b).

**Table 5:** List of oligosaccharides identified in F0s based on UHPLC-MS elution and fragmentation patterns

Peak no	Retention time (min)	*m*/*z* [M + 2AA]^−^	MS^2^ fragments^a^	Annotation^b^
1	7.67	624	**282**, 462, 444, 238, **300**, 204, 221, 220, 178, 202	H_3_
2	8.32	594	**282**, 238, 462, 204, **300**, 444, 414, 432, 178, 190, 202	H_2_P(H)
3	9.89	594	**300**, **282**, 462, 238, 204, 414, 432, 256, 178, 202, 176, 444, 191, 172, 498	H_2_P(H)
4	10.86	710	534, 648, 666, 576	P_3_U
4	10.86	786	383, **282**, **300**, 462, 444, 624, 238, 690, 384, 606, 221, 324, 750	H_4_
5	11.11	624	462, **282**, 204, 444, **300**, 463, 238, 221, 528, 342, 192	H_3_
6	12.08	710	534, 648, 666, 576	P_3_U/U^2^XX^c^
6	12.13	594	204, **300**, **282**, 462, 414, 252, 432, 221, 444, 256, 176, 270	H_2_P(H)
6	12.28	709	534, 576, 516, 402, 384	P_3_A
7	12.76	948	786, 624, 768, 828, 383, 462, 606, **282**	H_5_
8	13.16	564	432, **252**, **270**, 226, 176, 204, 414, 162, 160, 171, 402, 172, 208	HP_2_(P)
8	13.16	710	534, 648, 666, 578	P_3_U
9	13.44	754	564, 678, 722, 606, 710	HP_2_U_me_(H)
10	14.26	786	624, 462, 606, **282**, **300**, 383, 444	H_4_
11	14.55	723	691, 576, 534, 692, 516, 402, 384	P_3_A_me_
12	15.75	724	534, 648, 692, 576, 680	P_3_U_me_/U^4m2^XX^c^
13	16.61	691	576, 534, 516, 402, 604	P_3_A_u_
14	17.04	666	534, 516, 384, 226, 252, 402, **270**	P_4_
14	17.04	692	534, 648, 576, 630, 516	P_3_U_u_
14	17.04	724	534, 648, 692, 576, 680, 516	P_3_U_me_
15	17.88	578	402, 516, 534, **270**	P_2_U/U^2^X^c^
16	18.71	594	462, **300**, **282**, 238, 256, 204, 444, 178, 202, 176	H_2_P(H)
16	18.71	724	534, 648, 692, 576, 680	P_3_U_me_
17	20.83	591	559, 402, 444, **252**, 384, 472, **270**, 226	P_2_A
17	20.67	696	534, 402, **270**, 226, 516, **252**, 384	HP_3_(P)
18	22.13	592	402, 516, 560, 548, 444, **252**, 384	P_2_U_me_/U^4m2^X^c^
19	23.01	594	**282**, **300**, 238, 462, 191, 204, 178, 256, 202, 176, 172, 444, 498, 220	H_2_P(H)
20	23.58	724	534, 692, 648, 576	P_3_U_me_
21	23.94	534	384, 402, 162, 336, 204, 208, 226, 176, **252**, 160	P_3_
22	24.41	726	414, 576, 432, 594, 396, 238, 264, 558, **282**	H_2_P_2_(H)
23	28.24	828	666, 534, 648, 402, **270**	HP_4_(P)
24	28.87	534	402, 226, 466, **252**, **270**, 160, 176, 384	P_3_
25	29.16	666	534, 226, 402, **270**, 516, **252**, 384	P_4_
26	29.49	534	402, **270**, 226, 160, **252**, 384, 176, 204, 162	P_3_

The peak numbers are according to Fig. 3b

^a^The fragments are listed in the order of decreasing relative abundance. The first one is the base peak. Only fragments above 5% relative abundance are listed. Fragments containing the 2-AA label and the reducing end are printed in bold

^b^ *H* hexose, *P* pentose, *U* uronic acid, *U*_*me*_ 4-*O*-methyl uronic acid, *U*_*u*_ hexenuronic acid, *A* uronamide, *A*_*me*_ 4-*O*-methyl uronamide, *A*_*u*_ hexenuronamide. If pentosyl and hexosyl residues were present in an oligosaccharide, the reducing end is indicated in brackets

^c^Annotation based on heteroxylan nomenclature [15]

In MALDI-TOF MS, larger UAXOS (P_4_U_(me)_–P_7_U_(me)_) were identified (Additional file 1: Figure S1B(i)) in F0s. Using UHPLC-MS^*n*^, these oligosaccharides were also detected, but they were not in one of the major peaks. In SEC A3, oligosaccharides up to DP 10 (P_9_U_(me)_) were detected, along with oligosaccharides containing a glucuronosyl residue and a hexosyl residue (HP_4–7_U_me_). It was suggested that these oligosaccharides comprise glucurono-arabinoxylo-oligosaccharides, similar to the ones described in Verbruggen et al. [16]. This suggestion was supported by the glycosidic linkage composition for SEC A3 (Additional file 4: Table S2) which indicated that the proportion of branched xylosyl residue (18% of total xylosyl residue) was higher compared to that in the combined pool A3–A7 (5%).

**Fig. 4:**
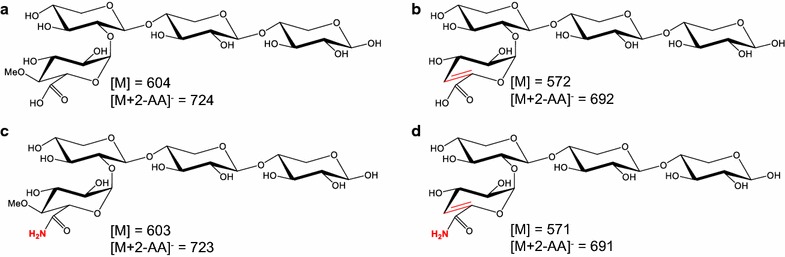
Variations of glucuronoxylo-oligosaccharides found in F0s, all containing a xylotriosyl backbone. The substituents are 4-*O*-me-glucuronosyl residue (**a**), hexenuronosyl residue (**b**), 4-*O*-me-glucuronamide residue (**c**) and hexenuronamide residue (**d**). [M], molecular weight of the molecule; [M + 2AA]^−^, molecular weight of the molecule after labelling by 2-aminobenzoic acid, detected by MS in negative mode

Besides UAXOS, an oligosaccharide composed of a xylotriosyl backbone and a hexenuronosyl residue linked to the non-reducing terminal xylosyl residue was detected (peak 14, *m*/*z* 692, Fig. 3b). The structure of this oligosaccharide is depicted in Fig. 4b, where variations of the glucuronosyl residue found in F0s are presented.

An unidentified oligosaccharide with *m*/*z* 626 was detected with MALDI-TOF MS (Fig. 1). The oligosaccharide was detected by UHPLC-MS^*n*^ in Peak 11 (*m*/*z* 723; Fig. 3b; Table 5). It was the most abundant oligosaccharide in SEC A11, eluting together with neutral tri- and tetrasaccharides. This pool was rich in xylosyl residues (Additional file 3: Table S1), with >50% of total xylosyl residue was 4-linked (Additional file 4: Table S2). The MS^2^ profile showed that this compound had a xylotriosyl backbone and could release methanol (−32 Da), just like U^4m2^XX, suggesting that the compound is similar to U^4m2^XX, but the glucuronosyl residue was modified. It has been described elsewhere that α-d-glucuronamide was present after treatment of glucuronoxylan-containing birch wood meal in ammonia [17]. The molecular weight of 4-*O*-methylglucuronamide is 207 and that of 4-*O*-methylglucuronic acid is 208. Hence, it is highly likely that the oligosaccharide in question (peak 11, Table 5) is a xylotriose with a 4-*O*-Me-glucuronamide residue (Fig. 4c). An oligosaccharide composed of a xylotriosyl backbone and a glucuronamide was present in peak 6 (*m*/*z* 709, Table 5). In peak 13, an oligosaccharide composed of a xylotriosyl backbone and a hexenuronamide substituent was detected (*m*/*z* 691; Fig. 4d).

#### Neutral oligosaccharides (SEC A8–A13)

Apart from UAXOS, F0s contained neutral oligosaccharides, which mainly eluted in SEC A8–A13. These neutral oligosaccharides (DP3–7) were rich in 6-linked, 4-linked and terminal glucosyl residues as well as terminal xylosyl residues (Tables 3, 4). Arabinosyl, mannosyl and galactosyl residues were present in small amounts.

#### Disaccharides (SEC A14–A15)

**Fig. 5:**
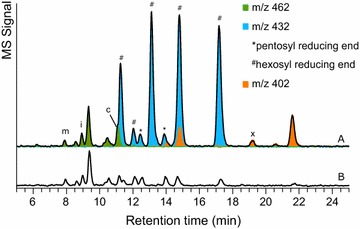
UHPLC-MS-extracted ion chromatograms of disaccharides in **a** 2-AA-labelled SEC pool A14 and **b** 2-AA-labelled SEC pool A14 after digestion by β-xylosidase from *A. awamori*. The *m*/*z* values of the detected disaccharides are indicated by the *colours*, as described in the legend. *m* mannobiose, *i* isomaltose, *c* cellobiose, *x* xylobiose

Disaccharides were abundant in F0s (Fig. 3a) and most disaccharides were isolated in SEC A14. First, the disaccharides were identified by their *m*/*z* values after labelling by 2-AA and analysis by UHPLC-MS. Disaccharides of hexoses have *m*/*z* value of 462, whereas disaccharides of pentoses have *m*/*z* 402. By comparison of the elution time and fragmentation patterns of the detected disaccharides to those of standard disaccharides, it was found that mannobiose (m), isomaltose (i), cellobiose (c) and xylobiose (x) were present (Fig. 5).

Nevertheless, most of the disaccharides detected were composed of a hexosyl and a pentosyl residues. The reducing end of these disaccharides could be identified based on the MS^2^ spectra. A hexosyl-reducing end with the 2-AA label has fragments with *m*/*z* 282 and 300, whereas a pentosyl-reducing end will produce fragments of *m*/*z* 252 and 270. It was found that most of the disaccharides consisted of a pentosyl residue attached to a hexosyl-reducing end. The major pentosyl and hexosyl residues in Pool A14–A15 were xylosyl and glucosyl residues, respectively (Table 4). Hence, it could be postulated that most of the disaccharides in the sample were xylosyl glucose, with different linkages. It was confirmed by the glycosidic linkage composition (Table 3) of SEC A14–A15 that showed 78% of the xylosyl residues were terminal residues. The results also showed the presence of glucosyl residues linked at position 2, 4 or 6. A small amount of 3-linked glucosyl residue was also present. Upon digestion with purified β-xylosidase from *Aspergillus awamori* [18], the disaccharides in all four major peaks were degraded, which indicated that the major disaccharides in Pool A14 had β-linkages. A disaccharide of xyloglucan, isoprimeverose (xyl(α1–6)glc), was present in small amounts (<1% (w/w) of Pool A14), as confirmed using high-performance anion exchange chromatography with pulsed amperometric detection (HPAEC-PAD) with the aid of standard isoprimeverose. Using UHPLC-MS^*n*^, isoprimeverose co-eluted with the β-linked xylosyl glucose at 14.7 min.

### Structural elucidation of water-soluble, less-polar oligosaccharides in F30s

**Fig. 6:**
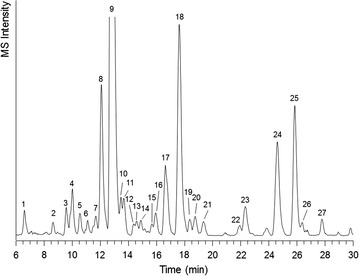
UHPLC-MS extracted ion chromatogram of oligosaccharides (*m*/*z* 501–2000) in 2-AA-labelled F30s. The peak numbers correspond to the data in Table 6

F30s was rich in xylosyl, glucosyl and arabinosyl residues (Table 2). The oligosaccharides in F30s were analysed using UHPLC-MS^*n*^ after labelling with 2-AA (Fig. 6). The results supported those obtained from MALDI-TOF MS that the majority of the oligosaccharides in F30s were mainly H_*x*_P_*y*_ oligosaccharides (Table 6). The most abundant oligosaccharide was H_3_P_2_, with *m*/*z* value of 888 after labelling with 2-AA (peak 9, Fig. 6).

**Table 6:** List of oligosaccharides identified in F30s based on UHPLC-MS elution and fragmentation patterns

Peak no.	Retention time (min)	*m*/*z* [M + 2AA]^−^	MS fragments^a^	Annotation^b^
1	6.59	786	624, 606, **282**, **300**, 238	H_4_
1	6.59	948	786, 768, **300**, 462, **282**, 444, 624, 606	H_5_
2	8.64	1356	1224, 1194, 1062, 1206, 930, 1092, 828, 1176, 1044	HP_8_ (P)
3	9.59	930	798, 780, 666, 402	P_6_
4	9.89	1050	918, 888, 900, 594, 576, 870, 756, 738, 353, **300**, 444, 1014, 930, 954	H_4_P_2_(H)
4	10.01	1062	930, 912, 798, 780, 666	P_7_
5	10.54	888	756, 594, 738, 576, **300**, **282**, 842	H_3_P_2_(H)
5	10.54	1050	756, 738, 918, 888, 870, 353, **300**, 900	H_4_P_2_(H)/LXG^c^
6	11.1	756	594, 353, **282**, 576, **300**, 624	H_3_P(H)/LG^c^
7	11.68	888	726, 738, 708, 414, 353, 756, 576, 432, 594, 660	H_3_P_2_(H)/LX^c^
8	12.08	1050	888,756, 738, 870, 918, 353	H_4_P_2_(H)/XLG^c^
9	12.8	888	756, 594, 738, 576, **300**, **282**	H_3_P_2_(H)/XXG^c^
9	12.8	1020	888, 870, 594, 726, 432, 576, **300**	H_3_P_3_(H)
10	13.48	1050	918, 900, 756, 738, 462, **300**, 444	H_4_P_2_(H)
11	13.66	930	798, 780, 648, 666, 534, 516	P_6_
12	14.37	960	798, 666, 828, 534, 780, 648	HP_5_(P)
13	14.48	930	798, 666, 780	P_6_
14	14.58	1050	918, 462, 756, 738, 900, 444	H_4_P_2_(H)
15	15.39	1092	798, 960, 666, 942, 534, 780, 648	HP_6_(P)
16	15.93	1050	918, 900, 738, 462, 756, 444, **300**	H_4_P_2_(H)
16	15.93	1182	1020, 888, 870, 1050, 1002, 432, 726	H_4_P_3_(H)/LXX^c^
17	16.63	798	666, 648	P_5_
17	16.63	902	756, 499, 594, 738, 367, **300**	H_3_PD(H)/FG^c^
17	16.76	1020	888, 726, 432, 708, 870, 414, 560, 474	H_3_P_3_(H)
18	17.63	726	414, 576, 432, 594, **282**, 238, 264, 558, 444	H_2_P_2_(H)
19	18.36	1020	888, 726, 708, 870, 432, 414	H_3_P_3_(H)
20	18.75	1196	1050, 888, 902, 884, 1032, 499, 756, 738, 1064, 870	H_4_P_2_D(H)/XFG^c^
21	19.35	798	666, 648	P_5_
22	21.93	1020	888, 726, 432, 708, 870, 952, 414, 560, 986	H_3_P_3_(H)
23	22.32	990	666, 828, 534, 810, 648, 402	H_2_P_4_(H)
24	24.6	828	666, 534, 648, 402, **270**, 516	HP_4_(P)
25	25.84	960	666, 828, 534, 810, 648, 402, 485	HP_5_(P)
26	26.38	666	534, 402, 226, 516, **270**, 252	P_4_
27	27.78	812	666, 534, 648	P_4_D

^a^The fragments are listed in the order of decreasing relative abundance. The first one is the base peak. Only fragments above 5% relative abundance are listed. Fragments containing the 2-AA label and the reducing end are printed in bold

^b^ *H* hexose, *P* pentose, *D* deoxyhexose. If pentosyl and hexosyl residues were present in an oligosaccharide, the reducing end is indicated in brackets

^c^Annotation according to xyloglucan nomenclature [20]

To be able to identify the hexosyl and the pentosyl residues in specific oligosaccharides, SEC was performed on F30s. The 17 pools collected could be categorised based on the molecular size of the oligosaccharides in the pools. SEC B1–B6 contained oligo-/polysaccharides with *m*/*z* >2000 Da that were not detected either by MALDI-TOF MS or by UHPLC-MS^*n*^. Based on MALDI-TOF MS results, SEC B7–B14 contained various oligosaccharides with *m*/*z* 800–2000, whereas B15–B17 were dominated by the most abundant pentasaccharide (H_3_P_2_; *m*/*z* 791) together with other DP4–6 oligosaccharides. Constituent monosaccharide and glycosidic linkage composition of individual pools (Additional files 3 and 4) as well as those of combined pools (Tables 3, 4) were used to elucidate the structures present in F30s.

#### Large (*m*/*z* > 2000) oligosaccharides (SEC B1–B6)

The large oligosaccharides in F30s were rich in xylosyl and arabinosyl residues (Table 4). The glycosidic linkage composition of these pools (Table 3) indicated the presence of 4-linked, 3,4-linked and 2,3,4-linked xylosyl residues as well as terminal arabinosyl residues. This finding suggested that these oligosaccharides were heavily substituted arabinoxylan.

#### Xyloglucan oligosaccharides (XGOS; SEC B11–B17)

**Fig. 7:**
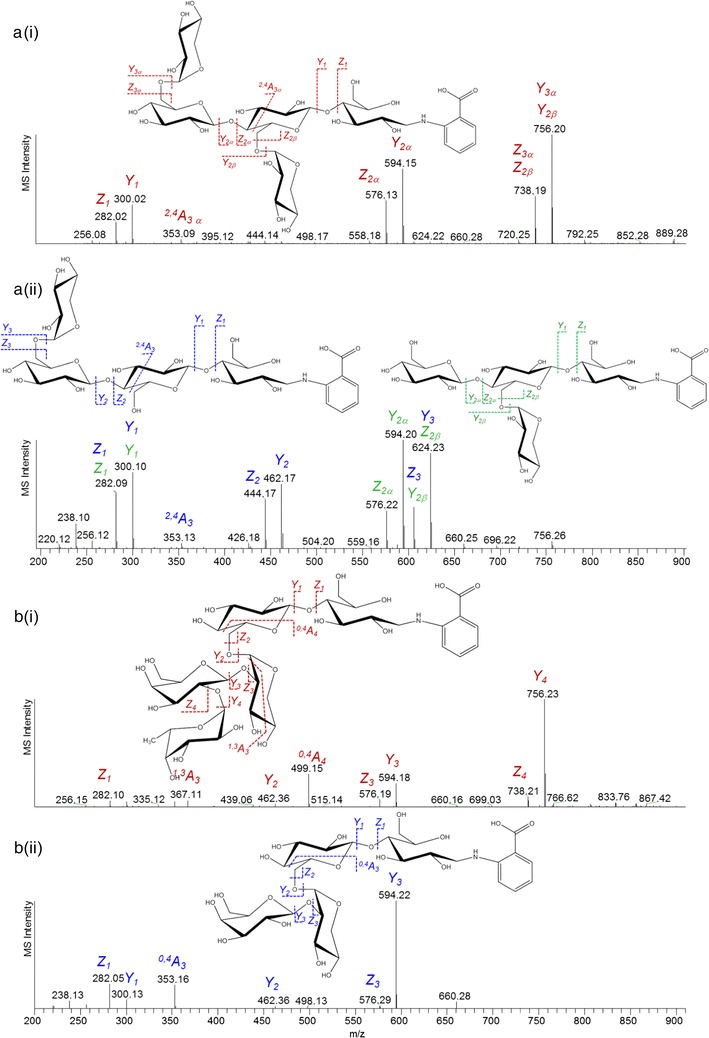
Negative mode ESI-MS^*n*^ fragmentation patterns of 2-AA-labelled xyloglucan pentasaccharides annotated as XXG (**a**) and FG (**b**). The fragments were annotated according to the nomenclature suggested by Domon, Costello [20]. A(i), MS^2^ fragmentation pattern of *m*/*z* 888; A(ii), MS^3^ fragmentation pattern of Y_3α_ (*blue*) and Y_2β_ (*green*) fragments (*m*/*z* 756). B(i), MS^2^ fragmentation pattern of *m*/*z* 902; B(ii), MS^3^ fragmentation pattern of Y_4_ fragment (*m*/*z* 756)

The most abundant oligosaccharide in F30s was annotated as H_3_P_2_ (peak 9, *m*/*z* 888). This compound was most abundant in SEC B16. The molar carbohydrate composition of this pool (Additional file 3: Table S1) consisted of 41 and 48% of glucose and xylose. More than 60% of the xylosyl residues were terminal, whereas the glucosyl residues were 4-linked (25%), 6-linked (42%), or 4,6-linked (29%) (Additional file 4: Table S2). Analysis of the MS^2^ and MS^3^ spectra of this compound indicated that the pentasaccharide had a glucotriosyl backbone with a xylosyl residue on two of the glucosyl residues (Fig. 7a). The structure corresponded with a xyloglucan building block annotated as XXG based on the nomenclature suggested elsewhere [19].

XGOS with DP >5 such as XXGG (*m*/*z* 1050; peaks 10, 14, 16; Fig. 6) and XXX (*m*/*z* 1020; peaks 17, 19, 22; Fig. 6) were present in SEC B11–B14, especially in SEC B13. For these oligosaccharides, however, there were isomers with similar MS^*n*^ fragmentation patterns eluting at different retention times, which complicated the annotation of the oligosaccharides.

Some XGOS were reported to contain galactosylated side chains [19]. Such oligosaccharides were identified by UHPLC-MS^*n*^ (Fig. 6; Table 6) in peaks 5 and 8 (LXG and XLG, *m*/*z* 1050), peak 6 (LG, *m*/*z* 756), peak 7 (LX, *m*/*z* 888) and peak 16 (LXX, *m*/*z* 1182). The galactosylated XGOS can be distinguished from the non-galactosylated ones by the prominent loss of a hexose (−162) and the more intense fragment ^0,4^A_*n*_ containing the disaccharide (xyl + gal) substitution (*m*/*z* 353). ^0,4^A_*n*_ fragments has been reported to be diagnostic for 1 → 6 linkage [21]. The presence of these oligosaccharides was supported by the constituent monosaccharide composition that shows that 6% of the carbohydrates in Pool B13 were galactosyl residues, 67% of which was identified as terminal galactosyl residues (Additional file 4: Table S2).

Some deoxy-hexose containing oligosaccharides were present in F30s (Table 6). These were annotated as fucosylated XGOS, such as FG (peak 17) and XFG (peak 20). The fragmentation pattern of FG in peak 17 is presented in Fig. 7b. The annotation of the fucosylated XGOS was facilitated by the abundance of ^0,4^A_4_ cross-ring fragment (*m*/*z* 499) containing the trisaccharide substitution, which was 1 → 6-linked to the xylosyl residue in the backbone [21]. The annotation was also supported by the presence of terminal fucosyl and 2-linked galactosyl residues in B13, although in small amounts (<1% mol/mol, Additional file 4: Table S2).

#### Other oligosaccharides in F30s (SEC B7–B14)

Apart from XGOS, UHPLC-MS^*n*^ show that F30s also contained hexo-oligosaccharides (H_4–5_; Table 6). Besides, F30s contain oligosaccharides rich in pentosyl residues (P_4_–P_7_, HP_4–8_). Some of these, like P_4_ and HP_4_ (peaks 24 and 26, Table 6) were similar to those detected in F0s (peaks 23 and 25, Table 5). By MALDI-TOF MS, larger oligosaccharides than those detected by UHPLC-MS^*n*^, including H_7–11_, P_6–12_ as well as oligosaccharides containing 4-*O*-methyl glucuronosyl residues (H_3_P_7–9_U_me_, H_4_P_7–8_U_me_ and H_5_P_7_U_me_) were detected in SEC B7–B14. These results indicated that the large molecules in F30s were heavily substituted heteroxylan.

## Discussion

The carbohydrate composition of DACS (Table 1) was similar to that of untreated corn stover [7], suggesting that no carbohydrates were lost during the dilute ammonia pretreatment. The degree of xylan substitution by arabinose and uronic acid was also comparable to literature for corn stover (0.14 and 0.09, respectively), with the assumption that all xylose, arabinose and uronic acid were components of heteroxylan [7]. Hence, the pretreatment did not cleave arabinosyl nor glucuronosyl residues from the xylan backbone. Ammonia pretreatment was expected to cleave ester-linked substitutions [22]. Indeed, although it was reported that acetyl and phenolic acid esters were present in corn stover [7], no acetylated oligosaccharides nor phenolic acid ester-containing oligosaccharide were detected in F-DACS.

Around 65% of the DACS carbohydrates were fermented after enzymatic hydrolysis. The remaining 10 and 25% (w/w) of the DACS carbohydrates after enzymatic hydrolysis and fermentation were present as water-soluble oligosaccharides and water-insoluble carbohydrates, respectively. A major fraction of the water-soluble oligosaccharides was composed of UAXOS. These oligosaccharides were degradation products of glucuronoxylan, which is the major hemicellulose in corn stover [7]. Most of the UAXOS analysed had a backbone of two or three xylosyl residues with the (4-*O*-methyl-) glucuronic acid attached at the non-reducing end. Such oligosaccharides can be cleaved by GH67 α-glucuronidases known to release the (4-*O*-methyl) glucuronic acid from the terminal xylosyl residue [23]. It was also shown that some of the glucuronosyl residues were modified into hexenuronosyl, glucuronamide, or hexenuronamide residues, most likely during the pretreatment in dilute ammonia. Hexenuronosyl residue can be formed by β-elimination of 4-*O*-methyl-glucuronosyl residue at alkaline pH [24], whereas glucuronamide may result from ammonolysis of ester bonds between a glucuronosyl residue and lignin [17]. Ester bonds between glucuronoxylan and lignin has been shown to be present, for example, in beech wood [25]. When both β-elimination and ammonolysis occurred to the same 4-*O*-methyl-glucuronosyl residue, a hexenuronamide residue was formed. Besides small (DP < 5) UAXOS, heavily substituted heteroxylan was suggested to be present in the remaining water-soluble fraction (Fs-DACS). It was reported that the substitutions in corn stover heteroxylan have a blockwise distribution, which may have resulted in recalcitrance to the hydrolytic enzyme mixture used [7].

Xyloglucan was the second major hurdle in the saccharification of DACS. XGOS were not expected to be abundant, because xyloglucan is known as a minor component in the cell wall of grasses [26]. Nevertheless, XGOS formed a major part of recalcitrant oligosaccharides in Fs-DACS. The most abundant XGOS present after hydrolysis and fermentation of DACS was XXG. This xyloglucan building block has been reported to be the most abundant xyloglucan building block in barley [27] and corn coleoptile [28]. Other XGOS, including galactosylated and fucosylated XGOS were also detected. In the literature, it was shown that grass xyloglucan does not contain fucosyl residues [26, 28]. It is possible, however, that the amount of fucosylated xyloglucan building blocks in the material used in the above-mentioned literature was below the detection limit. In this research, a large proportion of the carbohydrates was removed during fermentation or separated in several isolation steps; hence, the presence of such minor building blocks may have become apparent. XGOS contain xylosyl and glucosyl residues. These residues can be fermented into ethanol if they can be released as monosaccharides. Addition of α-xylosidase into the enzyme mixture is suggested to increase the fermentation yield of DACS.

Hexo-oligosaccharides also remained in the sample after hydrolysis and fermentation. Carbohydrates present in grass cell wall material that potentially produce hexo-oligosaccharides when partially degraded include mixed-linkage glucan, mannan and glucomannan [6]. Some large carbohydrates that were detected in the sample include pectin and arabinan, which were known as minor components in corn stover [6, 26] and not targeted by the enzyme mixture used for hydrolysis.

Besides oligosaccharides, disaccharides were found in significant amounts in the fermentation liquid. Most of the disaccharides were xylosyl glucose with β-linkages at different positions. Such disaccharides were not expected to be degradation products of the major polysaccharides in corn stover. The abundance and the variation of the disaccharides led to the hypothesis that they were produced by the enzymes used for hydrolysis. It was reported that transxylosylation activity is widely found in various fungal β-xylosidases [29]. It was intriguing, though, that most of the disaccharides were degradable by β-xylosidase from *A. awamori*. This result indicated that the β-xylosidase present in the enzyme mixture was inhibited by other compounds in the fermentation liquid.

## Conclusions

In this study, recalcitrant oligosaccharides present after hydrolysis and fermentation of DACS were identified. The majority of the identified oligosaccharides were parts of substituted heteroxylan. Small (DP < 5) UAXOS were abundant. Some of the recalcitrant glucuronosyl residues were modified to hexenuronosyl, (4-*O*-methyl)-glucuronamide or to hexenuronamide during pretreatment in ammonia. A second major group of oligosaccharides that were identified in this research were XGOS including galactosylated and fucosylated XGOS. The information about the recalcitrant structures present after fermentation can be used to fine tune the enzyme mixture used for hydrolysis. Based on the results of this research, in particular, addition of α-glucuronidase and α-xylosidase are expected to increase the release of xylose and glucose from DACS.

## Methods

When not specified, chemicals were of analytical grade (Sigma-Aldrich, Steinheim, Germany).

### Samples and sample preparation

Dilute ammonia-pretreated corn stover (DACS) and DACS suspension after hydrolysis and fermentation (F-DACS) were provided by DuPont Industrial Biosciences (Palo Alto, CA, USA). The hydrolysis was performed using an experimental enzyme cocktail containing enzymes to degrade cellulose and hemicellulose. The fermentation was performed using *Zymomonas mobilis* that can ferment glucose, xylose and arabinose [30]. Before extraction of soluble compounds, DACS (57% (w/w) solids) was thawed, freeze-dried and ball-milled (MM2000, Retsch, Haan, Germany). F-DACS was received as a slurry (16% (w/w) solids). Extraction of soluble solids from F-DACS was performed directly on the slurry after thawing.

### Isolation procedures

#### Extraction of soluble compounds

An aliquot of 20 g freeze-dried and milled DACS was mixed with 400 mL water. The soluble fraction was extracted at 65 °C for 24 h. The suspension was centrifuged (10,000×*g*, 20 °C, 30 min. The supernatant was collected, and the pellet was washed with water at 65 °C for 15 min followed by centrifugation (10,000×*g*, 20 °C, 30 min). The washing was performed three times, and the supernatant was combined with the first one. The combined supernatant (sDACS) and the pellet (iDACS) were freeze-dried.

Hydrolysed and fermented DACS (F-DACS) was thawed at 4 °C and centrifuged (10,000×*g*, 4 °C, 30 min. The supernatant was collected and immediately heated in a boiling water bath for 30 min. The pellet was washed with ice-cold water three times followed by centrifugation (10,000×*g*, 4 °C, 30 min). After every centrifugation, the supernatant was immediately heated in a boiling water bath for 30 min. During the heating of the supernatant, precipitate was formed, which was removed by filtration using filter paper (Schleicher & Schuell GmbH, Dassel, Germany). The precipitate was less than 0.1% (w/w) of the total solids of F-DACS and was not analysed further. Finally, the pellet was collected, suspended in water and heated in boiling water bath for 30 min. The filtered supernatant (Fs-DACS) and the pellet (Fi-DACS) were freeze-dried and stored for subsequent analyses.

#### C18 solid-phase extraction

Fs-DACS (1.2 g) was dissolved in 10 mL water and loaded on a C18 SPE column (35 cc, 10 g, Waters Corp., Milford, MA, USA). The SPE column was pre-activated using methanol (3 × 35 mL) and washed with water (3 × 35 mL). During sample loading, the flowthrough was collected. The column was washed with 100 mL water and the flowthrough during washing was combined with the sample flowthrough (F0). The C18-bound oligosaccharides were eluted with 80 mL 30% (v/v) methanol (F30). F0 was freeze-dried, whereas F30 was partially dried under a stream of air to remove the methanol, followed by freeze-drying.

#### Isolation of oligosaccharides in 67% (v/v) ethanol

Dried F0 (832 mg) was dissolved in 10 mL water, whereas dried F30 (289 mg) was dissolved in 5 mL water. Two parts of ethanol was added to one part of F0 or F30 solutions and mixed. After centrifugation, the supernatants (F0s and F30s) and the pellets (F0i and F30i) were separated, followed by drying under a stream of air.

#### Size exclusion chromatography (SEC)

F0s and F30s were dissolved in water (25 mg/mL) and the solutions were filtered (0.45 μm) before injection on SEC. For every SEC run, 4 mL solution was manually injected. In total, two runs and six runs were performed for F30s and F0s, respectively.

SEC was performed using an AKTA purifier (GE Healthcare, Piscataway, NJ, USA), equipped with a series of three columns packed with Superdex 30 HiLoad 26/60 (GE Healthcare) maintained at 35 °C, using water containing 0.5% (v/v) ethanol as eluent, as described elsewhere [31]. Fractions of 7 mL each were collected between 0.32 CV and 1.12 CV. The oligosaccharide profiles of each fraction after each run were analysed using MALDI-TOF MS. Fractions with similar oligosaccharide profiles were pooled, freeze-dried and re-dissolved in water at 5, 10, or 50 mg/mL depending on the amount obtained after freeze-drying. All pools were analysed for monosaccharide constituent composition by methanolysis and for oligosaccharide profiles by MALDI-TOF MS. Selected pools were also analysed for oligosaccharide profiles by UHPLC-MS^*n*^ after labelling with 2-AA and for glycosidic linkage composition.

### Analytical methods

#### Constituent monosaccharide composition

The constituent monosaccharide composition of DACS and samples after fractionation based on solubility in water (sDACS, iDACS, Fs-DACS and Fi-DACS) were analysed after pre-hydrolysis in 72% (w/w) sulphuric acid at 30 °C for 1 h followed by hydrolysis in 1 M sulphuric acid at 100 °C for 3 h. The uronic acids in the hydrolysate were quantified using an automated colorimetric m-hydroxydiphenyl assay [32]. Galacturonic acid solutions (12.5–100 μg/mL, calculated as anhydrous galacturonic acid) were used as standards. The neutral monosaccharides were derivatised to their alditol acetates as described elsewhere [33]. A mixture containing rhamnose, arabinose, xylose, galactose, glucose and mannose was used as standard. A separate standard was prepared for fucose. The alditol acetates were separated and quantified using gas chromatography with flame ionisation detector (Focus-GC-FID, Thermo Scientific, Waltham, MA, USA) using inositol as an internal standard. The column used was DB-225 (15 m × 0.53 mm id × 1 μm film thickness; Agilent Technologies, Santa Clara, CA, USA). The initial column temperature was 180 °C with 2 min holding time. The temperature was then increased to 210 °C with a ramp of 2 °C/min, followed by 5 min holding time. The injector and detector temperature was set at 220 °C. Helium was used as carrier gas with a constant pressure of 60 kPa. The analyses were performed in duplicate.

The constituent monosaccharide compositions of F0s, F0i, F30s, F30i and SEC pools were analysed after methanolysis in 2 M HCl followed by hydrolysis with 2 M TFA [34]. A standard mixture containing fucose, rhamnose, arabinose, xylose, glucose, galactose, mannose, glucuronic acid and galacturonic acid was treated in the same manner as for the samples and used for quantification. The monosaccharides were quantified using an ICS 5000 HPAEC-PAD with post-column addition (Thermo Scientific), equipped with a CarboPac PA-1 column (2 × 250 mm) and a guard column (2 × 50 mm; Thermo Scientific). The monosaccharides were separated as published elsewhere [35], the difference being a flow rate of 0.4 mL/min used instead of 0.3 mL/min. Glucuronic acid was used as a quantification standard for both glucuronic acid and 4-*O*-me-glucuronic acid.

#### Glycosidic linkage composition

To analyse the glycosidic linkage composition, aliquots of samples containing 50 μg of carbohydrates was dried under a stream of nitrogen. The samples were methylated, hydrolysed, reduced and acetylated according to the procedure described elsewhere [36]. After acetylation, the dichloromethane used to extract the partially methylated alditol acetates (PMAA) was evaporated. The PMAA were washed twice with acetone and re-dissolved in 50 μL ethyl acetate for analysis. The separation was performed using Gas Chromatography (TRACE GC Ultra, Thermo Scientific) equipped with a Rtx-35MS column (30 m, ID 0.25 mm; Restek, Bellefonte, PA, USA). A 52-min temperature gradient starting from 120 to 250 °C was applied, followed by a holding time of 5 min. The eluted compounds were monitored using a Mass Spectrometer (DSQ II, Thermo Scientific) set in positive mode, with *m*/*z* range of 50–450.

For identification, the retention time and the MS^2^ profiles of the detected compounds were compared with the profiles obtained from the standards, as well as with the PMAA MS^2^ database published by Complex Carbohydrate Research Center (Georgia, USA) [37]. The standards used include maltose, isomaltose, amylopectin (Sigma-Aldrich), isoprimeverose, laminaribiose, xylobiose, XGOS mixture, galactobiose, mannotriose, wheat arabinoxylan (Megazyme, Bray, Ireland) and soluble soy polysaccharides (Fuji Oil Co. Ltd., Ibaraki, Japan). The equipment control and data analysis were performed using Thermo Xcalibur software (version 2.2 SP1.48).

#### Labelling and cleaning

For labelling, aliquots containing ~125 μg of carbohydrates for mixtures or ~0.3 μmol for pure oligosaccharides were dried under vacuum and labelled as described elsewhere [38]. The labelling was performed in a solution of 30% (v/v) acetic acid in DMSO containing 0.35 M 2-aminobenzoic acid (2-AA; Sigma-Aldrich Chemie, GmbH, Steinheim, Germany) and 1 M 2-picoline borane (Sigma-Aldrich). The mixture was incubated at 65 °C for 2 h.

Excess label and reducing agent were removed using a modification of a cleaning procedure described elsewhere [39]. The sample was cooled to room temperature, mixed with 50 μL water and 900 μL acetonitrile, then loaded onto a Bond-Elut cellulose SPE column (Agilent, Santa Clara, CA, USA) that was preconditioned with 4 mL water and washed with 6 mL 90%(v/v) acetonitrile. After sample loading, the column was washed with 6 mL 90% (v/v) acetonitrile to remove excess label. Labelled oligosaccharides were eluted with three aliquots of 500 μL water, dried under vacuum and dissolved in 100 μL water. The sample was diluted ten times before analysis with UHPLC-MS^*n*^.

#### UHPLC-MS^*n*^

UHPLC-MS^*n*^ was performed using an Accela UHPLC system (Thermo Scientific) equipped with Acquity BEH C18 column (2.1 × 150 mm, particle size 1.7 μm) preceded with a guard column (Waters Corp., Milford, MA, USA). The column was kept at 25 °C, and the samples were kept at 20 °C. The needle was flushed and washed with water before and after every injection. The eluents were water (A) and acetonitrile (B), each containing 0.1% formic acid (both were UHPLC-grade; Biosolve, Valkenswaard, The Netherlands). The injection volume was 2 μL. For F0s and SEC pools from F0s, the separation started with an isocratic elution of 8% B for 15 min, followed by a gradient from 8 to 10% B in 10 min. Afterwards, a gradient of 10–20% B in 10 min was applied. After each analysis, the column was cleaned with 50% B for 5 min, followed by re-equilibration at 8% B for 8 min. The flow rate was 300 μL/min.

For F30s and SEC pools from F30s, another gradient was applied. The flow rate was 200 μL/min. The elution was started with an isocratic elution of 10% B for 20 min, followed by a gradient from 10 to 15% B in 10 min, cleaning at 50% B for 5 min and re-equilibration at 10% B for 8 min.

The elution of 2-AA-labelled compounds was monitored by a photodiode array (PDA; Thermo Scientific) set to monitor the absorbance at 254 nm [40]. The compounds were also detected by a MS (Velos Pro, Thermo Scientific) set at negative mode. The source heater temperature, capillary temperature and the ion source voltage were set at 225 °C, 350 °C and −4.5 kV, respectively. Detection mass range was set to range between 400 and 2000 Da. MS^*n*^ was performed using a normalised collision energy of 35 (arbitrary units). To obtain MS^*n*^ spectra of co-eluting compounds, dynamic exclusion was set with an exclusion list size of 25 and exclusion duration of 15 s. Dynamic exclusion was terminated earlier when there were three consecutive scans resulting in noise-level spectra. Thermo Xcalibur software (version 2.2 SP1.48) was used for equipment control and the data analysis.

#### Oligosaccharide profiling using MALDI-TOF mass spectrometry

MALDI-TOF mass spectrometry was performed using an UltrafleXtreme TOF (Bruker Daltonics, Bremen, Germany). SEC fractions were analysed directly. Other samples were dissolved at 1 mg/L in water. Sample solutions that may contain salts were pretreated by incubation with Dowex AG 50 W-X8 resin (Bio-Rad, Hercules CA, USA) for 30 min at room temperature. Sample preparation and analysis were performed as described elsewhere [31].

#### Molecular mass distribution (HPSEC-RI)

Molecular mass distribution was analysed using high-performance size exclusion chromatography with refractive index detection (HPSEC-RI). Freeze-dried samples were dissolved at 2.5 mg/mL in water and centrifuged (10,000×*g*, 5 min, 20 °C) to remove any insoluble material. An aliquot of 10 μL of the supernatant was analysed as described elsewhere [7]. The elution temperature used here was 55 °C instead of 40 °C.

## Additional files

**Additional file 1. Figure S1.** MALDI-TOF mass spectra of Fs-DACS fractions after separation using C18 SPE (A) and after isolation in 67% (v/v) ethanol (B).

**Additional file 2. Figure S2.** Molecular mass distribution of compounds in Pool A1 and A2 obtained after high performance size exclusion chromatography of F0s.

**Additional file 3: Table S1.** Monosaccharide composition of pools obtained after SEC of F0s (A1-A17) and F30s (B1-B19).

**Additional file 4: Table S2.** Glycosidic linkage composition of selected SEC pools after separation by SPE C18 and isolation in 67% (v/v) ethanol.

## Abbreviations

DACS: dilute ammonia-pretreated corn stover
F-DACS: hydrolysed and fermented DACS
F0/F30: a fraction of Fs-DACS, eluted with water/30% (v/v) methanol through C18 SPE column
F0i/F30i: fractions of F0/F30 that was insoluble in 67% (v/v) ethanol
F0s/F30s: fractions of F0/F30 that was soluble in 67% (v/v) ethanol
HPAEC-PAD: high-performance anion exchange chromatography, with pulsed amperometric detection
(HP)SEC: (high-performance) size exclusion chromatography
iDACS/Fi-DACS: water-insoluble fraction of DACS/F-DACS
MALDI-TOF: matrix-assisted laser desorption ionisation time of flight
MS: mass spectrometry
PMAA: partially methylated alditol acetates
sDACS/Fs-DACS: water-soluble fraction of DACS/F-DACS
SPE: solid-phase extraction
UAXOS: glucurono-xylooligosaccharides
UHPLC-MS: ultra high-performance liquid chromatography
XGOS: xyloglucan oligosaccharides
