# Supplementary material for: Characterisation of non-degraded oligosaccharides in enzymatically hydrolysed and fermented, dilute ammonia-pretreated corn stover for ethanol production
Source: Biotechnol Biofuels. 2017 May 2;10:112. doi: 10.1186/s13068-017-0803-3 (PMC5414315; doi:10.1186/s13068-017-0803-3)
Supplement: Supplementary file 1 — Additional file 1. Figure S1. MALDI-TOF mass spectra of Fs-DACS fractions after separation using C18 SPE (A) and after isolation in 67% (v/v) ethanol (B). [file 13068_2017_803_MOESM1_ESM.pdf]

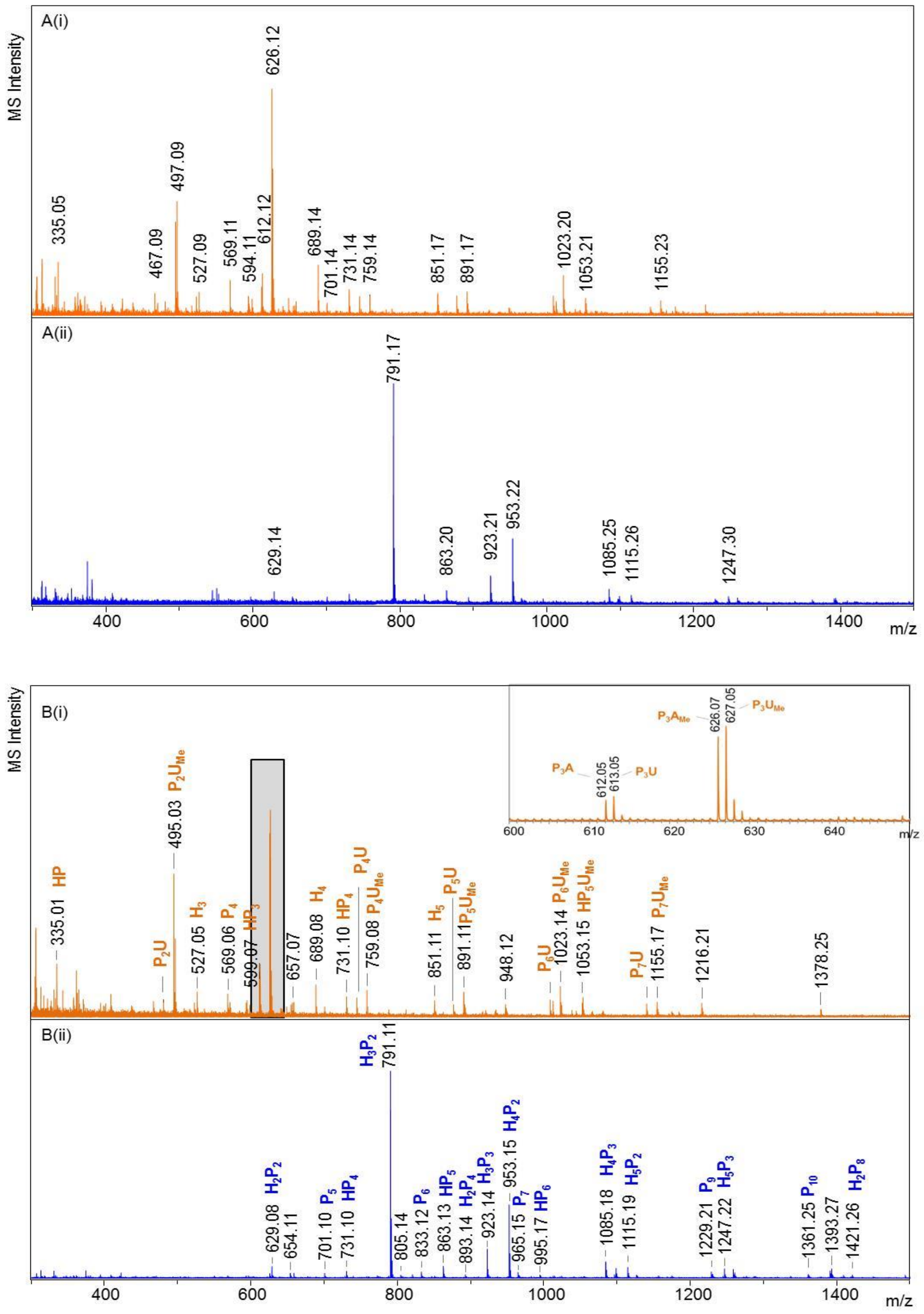

Figure S1. MALDI-TOF mass spectra of Fs-DACS fractions after separation using C18 SPE (A) and after isolation in 67 % (v/v) ethanol (B). The ions were detected in positive mode as their sodium adducts $(M+23)^+$. (i): water-eluted fraction (A: F0, B: F0s), (ii): 30 % (v/v) methanol-eluted fraction (A: F30, B: F30s). B(Inset: Zoom in of m/z range 600-650. H: hexose, P: pentose, U: uronic acid, U_{me}: 4-*O*-methyl uronic acid, A: uronamide, A_{me}: 4-*O*-methyl uronamide.
