# Supplementary material for: Characterisation of non-degraded oligosaccharides in enzymatically hydrolysed and fermented, dilute ammonia-pretreated corn stover for ethanol production
Source: Biotechnol Biofuels. 2017 May 2;10:112. doi: 10.1186/s13068-017-0803-3 (PMC5414315; doi:10.1186/s13068-017-0803-3)
Supplement: Supplementary file 2 — Additional file 2. Figure S2. Molecular mass distribution of compounds in Pool A1 and A2 obtained after high performance size exclusion chromatography of F0s. [file 13068_2017_803_MOESM2_ESM.pdf]

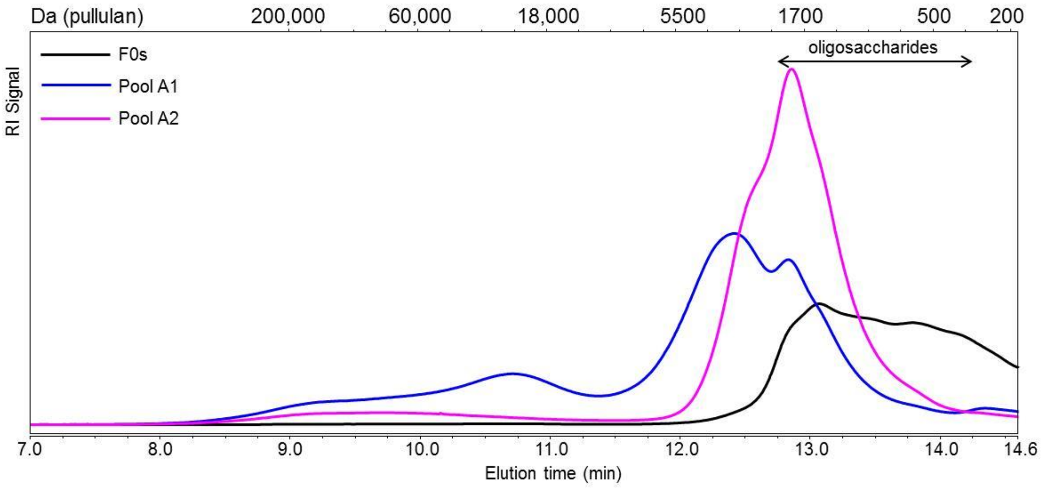

Figure S2. Molecular mass distribution of compounds in Pool A1 and A2 obtained after high performance size exclusion chromatography of F0s.
