# Supplementary material for: Characterisation of non-degraded oligosaccharides in enzymatically hydrolysed and fermented, dilute ammonia-pretreated corn stover for ethanol production
Source: Biotechnol Biofuels. 2017 May 2;10:112. doi: 10.1186/s13068-017-0803-3 (PMC5414315; doi:10.1186/s13068-017-0803-3)
Supplement: Supplementary file 3 — Additional file 3: Table S1. Monosaccharide composition of pools obtained after SEC of F0s (A1-A17) and F30s (B1-B19). [file 13068_2017_803_MOESM3_ESM.pdf]

Table S1. Monosaccharide composition of pools obtained after SEC[a] of F0s (A1-A17) and F30s (B1-B19)

SEC Pool	Mol %[b]								
	fuc	ara	rha	gal	glc	xyl	man	galA	glcA
A1	1	19	7	14	33	14	2	7	3
A2	0	7	2	7	28	40	3	7	7
A3	0	10	1	5	24	48	2	3	6
A4	0	9	0	3	15	61	2	3	7
A5	0	4	0	2	12	68	2	4	8
A6	0	2	0	1	9	71	3	4	10
A7	0	2	0	2	10	64	7	3	12
A8	0	2	0	2	18	53	6	3	16
A9	0	3	0	5	25	39	5	2	21
A10	0	8	0	7	23	44	4	1	13
A11	0	8	0	4	23	54	3	2	6
A12	0	5	0	3	38	42	7	1	4
A13	0	4	0	3	48	37	5	1	2
A14	0	5	0	1	38	49	4	1	1
A15	0	6	0	3	25	59	4	1	1
B1	0	36	3	15	5	36	2	1	2
B2	1	31	1	13	6	44	2	1	2
B3	0	27	1	9	6	51	1	1	3
B4	0	22	1	8	12	49	2	1	4
B5	0	22	1	11	18	41	2	1	3
B6	0	22	1	10	18	45	2	1	1
B7	0	21	1	8	17	48	2	1	1
B8	0	20	1	8	19	49	1	1	1
B9	0	20	1	7	19	51	1	1	1
B10	1	20	1	7	19	51	1	0	1
B11	1	17	0	8	19	52	1	1	1
B12	1	9	0	8	27	51	1	1	1
B13	1	9	0	8	30	49	1	0	1
B14	1	9	0	8	30	49	1	1	1
B15	1	8	0	7	32	50	1	0	1
B16	0	6	0	3	41	48	1	0	1
B17	0	7	0	3	36	50	2	0	1

[a]Water containing 0.5 % (v/v) ethanol was used as eluent. Negatively charged oligosaccharides, such as glucuronoxylo-oligosaccharides did not interact with the column material used (Superdex 30) and eluted earlier than neutral oligosaccharides of the same DP. Similar observation has been reported for Bio-Gel P2 size exclusion chromatography [36].

[b]fuc: fucose, ara: arabinose, rha: rhamnose, gal: galactose, glc: glucose, xyl: xylose, man: mannose, galA: galacturonic acid, glcA: glucuronic acid
