# Supplementary material for: Characterisation of non-degraded oligosaccharides in enzymatically hydrolysed and fermented, dilute ammonia-pretreated corn stover for ethanol production
Source: Biotechnol Biofuels. 2017 May 2;10:112. doi: 10.1186/s13068-017-0803-3 (PMC5414315; doi:10.1186/s13068-017-0803-3)
Supplement: Supplementary file 4 — Additional file 4: Table S2. Glycosidic linkage composition of selected SEC pools after separation by SPE C18 and isolation in 67% (v/v) ethanol. [file 13068_2017_803_MOESM4_ESM.pdf]

Table S2. Glycosidic linkage composition[a] of selected SEC pools after separation by SPE C18 and isolation in 67 % (v/v) ethanol

	mol %													
Linkage[b]	A3	A5	A7	A11	A12	A14	A15	B4	B7	B9	B11	B13	B16	B17
t-ara	7	2	1	1	1	1	1	12	12	11	10	2	2	3
2-ara	1	0	0	4	1	0	1	4	4	5	4	3	1	1
3-ara	3	1	0	2	1	0	0	3	3	3	2	1	0	0
5-ara	1	0	0	0	0	0	1	3	1	1	1	0	0	1
2,5-ara	4	2	1	0	0	0	0	3	3	3	2	2	0	0
**Total ara**	**15**	**6**	**3**	**7**	**3**	**2**	**3**	**25**	**24**	**23**	**19**	**9**	**4**	**6**
t-xyl	13	12	8	12	21	40	54	14	13	13	14	21	31	34
2-xyl	6	14	20	10	5	2	13	5	4	4	7	9	4	4
3-xyl	1	1	0	4	3	1	4	1	1	0	1	2	3	3
4-xyl	27	47	48	34	17	6	6	19	18	19	18	16	8	11
2,3-xyl	2	1	1	0	0	0	0	2	2	1	1	1	1	0
3,4-xyl	6	3	1	0	0	0	0	8	8	7	6	2	0	1
2,3,4-xyl	3	1	0	0	0	0	0	4	4	5	3	1	0	1
**Total xyl**	**58**	**80**	**78**	**61**	**45**	**50**	**77**	**52**	**49**	**49**	**50**	**52**	**46**	**53**
t-fuc	0	0	0	0	0	0	0	0	0	0	1	0	0	0
**Total fuc**	**0**	**0**	**0**	**0**	**0**	**0**	**0**	**0**	**0**	**0**	**1**	**0**	**0**	**0**
t-man	0	0	5	2	4	4	0	0	0	0	0	0	0	0
4-man	0	0	0	1	3	1	1	0	0	0	0	0	0	1
**Total man**	**0**	**0**	**6**	**2**	**6**	**5**	**1**	**0**	**0**	**0**	**0**	**0**	**0**	**1**
t-glc	3	2	2	7	10	11	1	10	10	9	8	2	1	3
2-glc	0	0	1	8	2	11	5	1	1	2	2	1	0	1
3-glc	0	0	0	0	1	2	1	0	0	1	0	0	0	1
4-glc	1	2	1	4	12	10	6	1	3	5	4	9	12	7
6-glc	16	7	5	5	17	7	4	5	6	5	6	11	20	19
4,6-glc	0	0	0	1	1	0	0	0	0	1	3	7	14	7
3,6-glc	1	0	0	0	0	0	0	0	0	0	0	0	0	1
**Total glc**	**22**	**12**	**10**	**25**	**42**	**42**	**17**	**18**	**22**	**22**	**24**	**32**	**48**	**37**
t-gal	2	1	1	3	2	1	1	3	3	3	4	4	1	1
4-gal	1	0	0	0	0	0	0	1	1	0	0	0	0	0
2-gal	0	0	0	0	0	0	0	0	0	0	1	1	0	0
3-gal	0	0	0	0	0	0	0	0	0	1	0	1	0	0
**Total gal**	**3**	**1**	**1**	**4**	**2**	**1**	**1**	**4**	**5**	**5**	**6**	**6**	**1**	**1**
**Total**	**98**	**99**	**97**	**99**	**99**	**99**	**99**	**99**	**99**	**99**	**99**	**99**	**100**	**99**

[a]Only neutral glycosyl residues were analysed. A glycosyl residue at the reducing end resulted in the same partially methylated alditol acetates as an internal glycosyl residue.

[b]t: terminal, rha: rhamnose, ara: arabinose, xyl, xylose, man: mannose, gal: galactose, glc: glucose.
